# Beyond Tissue replacement: The Emerging role of smart implants in healthcare

**DOI:** 10.1016/j.mtbio.2023.100784

**Published:** 2023-08-29

**Authors:** Elena Abyzova, Elizaveta Dogadina, Raul D. Rodriguez, Ilia Petrov, Yuliana Kolesnikova, Mo Zhou, Chaozong Liu, Evgeniya Sheremet

**Affiliations:** a Tomsk Polytechnic University, Lenin ave. 30, Tomsk, Russia, 634050; b Institute of Orthopaedic & Musculoskeletal Science, University College London, Royal National Orthopaedic Hospital, Stanmore, HA7 4LP, UK

**Keywords:** Smart implants, Implantable electronics, Sensors and stimulation devices, Implant materials, Remote health monitoring, Continuous monitoring

## Abstract

Smart implants are increasingly used to treat various diseases, track patient status, and restore tissue and organ function. These devices support internal organs, actively stimulate nerves, and monitor essential functions. With continuous monitoring or stimulation, patient observation quality and subsequent treatment can be improved. Additionally, using biodegradable and entirely excreted implant materials eliminates the need for surgical removal, providing a patient-friendly solution. In this review, we classify smart implants and discuss the latest prototypes, materials, and technologies employed in their creation. Our focus lies in exploring medical devices beyond replacing an organ or tissue and incorporating new functionality through sensors and electronic circuits. We also examine the advantages, opportunities, and challenges of creating implantable devices that preserve all critical functions. By presenting an in-depth overview of the current state-of-the-art smart implants, we shed light on persistent issues and limitations while discussing potential avenues for future advancements in materials used for these devices.

## Graphical abstract

**Figure:**
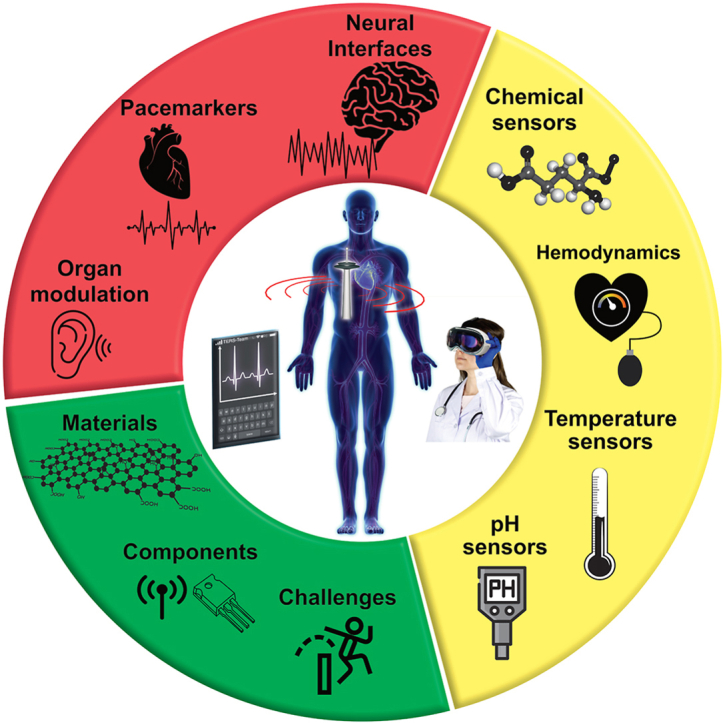

## 1 Introduction

Smart implants are transforming the healthcare industry and offering new opportunities to enhance the quality of life for individuals. Throughout history, people have used various dental implants, such as sea mussel shells, metal, stone, and animal bones. The first implants used were finger and limb prosthetics, but over the past seven decades, implant technology has progressed tremendously, delivering outstanding results and exceeding patient expectations. Smart implants are becoming increasingly important with the growing trend toward human augmentation. These devices have the potential to not only improve the lives of people with congenital diseases, acquired diseases, and the elderly but also to extend life expectancy, correct facial and body imperfections, and even enable movement for those with disabilities [1].

**Fig. 1:**
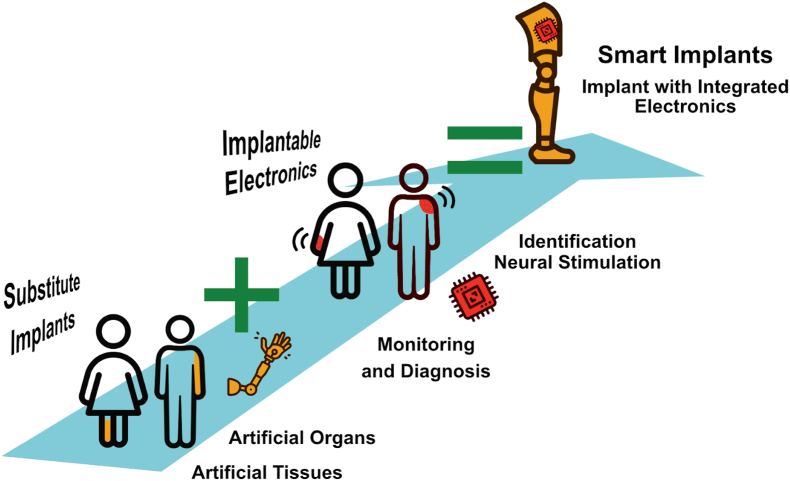
Schematic representation of the categories of implantable devices: substitute implants, implantable electronics, and smart implants.

Implants can be divided into two categories: those that substitute a human body part, tissue, or organ, and those that are "smart" - augmented with electronic components, which add additional functions that were not initially present in the body or lost due to an accident or disease. For example, smart implants can stimulate nerves, monitor essential body functions, support internal organs, and actively contribute to patients’ recovery and well-being. (Fig. 1).

**Substitute implants** are divided into artificial tissues and artificial organs. Artificial tissues are designed to replace damaged human organ parts, such as skin, bones, cartilage, and vessels, by patching only the necessary part through tissue engineering [2]. Two research areas are rapidly developing in tissue engineering: cells and scaffolds. Cells multiply and differentiate into tissues, while scaffolds are three-dimensional structures that support cell growth [3]. Tissue engineering has shown promise in treating heart-related diseases using implantable artificial blood vessels [4], injectable gels [4,5], and cardiac patches [6], as well as in creating engineered heart tissue [7,8]. Tissues can also successfully replace parts of organs such as the esophagus [9], liver [10], tympanic membrane [11], *etc*. Such materials can be loaded with drugs preventing infection and reducing pain syndrome [12] or with antitumor agents [13].

Organ shortage problems caused by the high demand for organ transplantation and the limited donor number can be solved by creating fully functional whole organs [11,14]. In addition to the projected increase in life expectancy and prevalence of chronic diseases, the need for organ replacements is expected to rise [15]. Developments on bionic organ creation are also underway, but at the moment, all models work only in laboratory conditions. Recently there have been works dedicated to the design of a functional artificial heart, lung, and pancreas [16] along with bionic hearts [17], skin [18], ears [19], urethral, and bladder sphincters. Separately, the study of artificial lymphoid organs, such as lymph nodes and spleen, is evolving [20].

These bioelectronic devices must meet the functional requirements of the body parts they are replacing while avoiding inflammation, toxicity, or breakdown and providing improved quality of life. There may also be pain at the implant site, which could mandate removal. Some implants, for instance, scaffolds for bone growth or screws used to fix broken bones, may become redundant after serving their purpose. Metallic implants can also pose a challenge for diagnostic procedures such as MRI [21]. The need for removal depends on the implant's location and composition, with some studies showing long-term pain symptoms after removing tibial nails [22]. However, it is still an open question whether to remove the implant if there are no complaints from the patient. There are also issues with implant survival rate, allergic reactions, and complications detected too late. Besides, long-term exposure to abiotic components near organs can trigger adverse immune responses [23].

Different types of **implantable electronics**, also known as "body-machine interfaces,” collect information about vital physiological indicators in the body and individual organs or the state of the implant itself, transforming it into readable signals. Several recent reviews have addressed this subject, such as those published in Refs. [[24], [25], [26]]. These devices use neurotechnology, electronics, and micro- and nanoscale interactions to enable body-machine interconnection and improve medical diagnosis and treatment. For instance, implantable identification tags can replace keys, cards, and medical records [20,27]. Monitoring implants can track crucial indicators such as pH, temperature, and bacteriological parameters related to infection, while wireless access to implant data opens up possibilities for advanced applications like drug delivery systems and prosthesis control [[28], [29], [30], [31]]. For instance, microfluidic lab-on-a-chip devices offer controlled drug release to target locations at specific times [32]. The same technological solutions can be used for neural stimulation implants to treat various diseases and conditions, such as Parkinson's disease, essential tremors, and pain syndromes [[33], [34], [35], [36]].

Besides fulfilling stringent safety and efficacy requirements, smart implants must perform mechanical, electronic, or sensing functions. They may consist of moving parts such as membranes, coils, or capacitor electrodes, other sensing elements such as electrochemical electrodes, or other electronic components comprised of precisely defined conducting, semiconducting and dielectric areas, as well as components powering the device or transmitting signals (see Section 5 for more details). Specific challenges in this domain encompass the development of strategies to mitigate fibrous tissue encapsulation, which can hinder direct contact between the sensor and the surrounding tissue, and ensuring the stability of the materials employed.

This review explores the possibility of augmenting substitute implants with electronic components to produce **smart implants** and the challenges and opportunities of creating implantable technology that preserves all critical functions. Here, we define **smart implants as devices that replace tissue or an organ and, at the same time, carry additional monitoring, diagnostic, or therapeutic function** [37,38]. These implants hold the potential to provide valuable health insights, anticipate potential complications, and allow for prompt intervention. As noted by Veletic et al. [26] in their review on implantable sensors, the integration of substitution, sensing, and stimulation functionalities within a single implantable device is still in its early stages of development. In this context, our discussion begins with a focus on well-established implantable sensing and stimulating electronic devices. Subsequently, we explore possibilities and challenges associated with their integration into substitute implants, aiming to pave the way for advanced implantable solutions. The main focus of this review is to address the critical challenge of achieving a balance between functionality and safety when integrating implantable electronic devices, their functions, and the associated materials. In this regard, the review aims to provide insights into two fundamental questions:

1. Are there substantial benefits in integrating implantable electronics into substitute implants that justify the risks associated with implantation?
2. What technological or medical barriers currently hinder the widespread integration of electronics into substitute implants?

By exploring these questions, we aim to shed light on the potential advantages, limitations, and areas for further development in implantable electronic devices, ultimately contributing to the advancement of safe and effective substitute implant technologies.

In this review, we begin with a brief introduction to the components of implantable electronics. We then delve into a comprehensive examination of sensing and stimulating components, providing an in-depth analysis of their applications, challenges, and advantages [39,40]. Additionally, we explore the materials employed in fabricating substitute implants and implantable electronics. Finally, we conclude the review by discussing the integration of electronic components into substitute implants, leading to the development of smart implants. It is important to note that this review specifically excludes drug delivery systems, actuators, and complex mechanical systems, as our focus is primarily on electronic components.

Smart implants require functional components for monitoring and stimulation functions to enhance healthcare quality, streamline patient treatment, and reduce visits to medical institutions [37]. These components include a sensitive or active element, a data transmission circuit, and an energy source (if the implant is active). The sensitive/active element plays a crucial role in the implant's function, either converting information into electrical signals for processing (sensitive element) or impacting the organ or tissue through electrical, mechanical, or chemical action (active component). For example, electrodes serve as an active element in neural interfaces by applying a potential or current to tissues [41], while electrochemical electrodes act as tissue-electronics interfaces in monitoring [42], and force and pressure sensors measure mechanical loads [40,43].

Before proceeding to the specific examples, it must be noted that implantation is an invasive procedure that leads to tissue damage, potential nerve damage, risk of infection, *etc*. The tissue damage can be minimized by optimizing the implant size and shape [44]. Moreover, a body perceives an implant as a foreign body that triggers an inflammatory response. This effect can be minimized by using a biocompatible material with the closest mechanical properties to body tissues [45]. Biocompatibility is improved by employing coatings [46]. Anti-inflammatory compounds, such as drugs or molecules with anti-inflammatory properties, can be applied to the implant's surface to reduce inflammation. Adhesive proteins or bioactive molecules promote better implant integration with the surrounding tissues [46]. Besides, there are also various medical treatments and solutions to mitigate inflammatory responses linked to implants. These interventions encompass the administration of anti-inflammatory drugs [47], utilization of immunomodulatory therapies [48], implementation of surface modifications [49], employment of physical barriers [50], and application of cryotherapy techniques [[51], [52], [53]]. When selecting and implementing these treatments, careful consideration should be given to the specific implant, target tissue, and desired outcome. Implant robustness is also crucial since its fracture, leak, or dislocation can cause serious toxicity and tissue damage.

## 2 Components of implantable electronics

The operation of active elements or measurements in implantable devices requires a power source, but unfortunately, this requirement imposes limitations on the minimum size of the implant [54]. Smart implants often rely on batteries to power their "smart" components and data transmission. Researchers are actively developing long-term, safe, and biodegradable batteries for use in smart implants [55,56]. Batteries offer advantages such as independent power, portability, and reliability. However, they also have drawbacks, including limited operating time, size and volume constraints, and the need for recharging or replacement. To overcome these limitations, alternative approaches are being explored, such as harvesting energy from the body itself or from various environmental sources. These energy harvesting methods eliminate the need for battery replacement and instead derive power from natural or artificial sources [57]. Examples of such energy sources include piezoelectric and triboelectric nanogenerators and thermoelectric generators [58]. For instance, Kim et al. developed a flexible energy harvester based on the piezoelectric effect, which generated energy from the contractions of a pig's heart [59,60]. Another example is the implantable enzymatic biofuel cell reported by Lee et al., which converts chemical energy into electrical energy using enzymes and microorganisms through electrocatalysis on electrodes for brain stimulation [61]. Recently, a thermally sterilizable glucose fuel cell with the highest power density was also reported [62].

Another strategy to power the device is to supply external energy wirelessly through inductive [63], capacitive [64], microwave [65], optical, or radio frequency [59] transmission. In inductive coupling, instead of batteries, it is proposed to use electric coils embedded in the body and the implant to receive energy from another coil outside [66]. The frequency used for power transmission varies depending on the tissue type between the external and internal components and the desired data transfer rate [67], with lower frequencies reducing losses but higher frequencies increasing the transfer rate. Most commercially available implantable devices use higher frequencies to improve the data transfer rate. Thus, the frequency range for different implant types ranges from the kilohertz range (e.g. 43.4–175 kHz for neural interfaces) up to the megahertz range (e.g. 5–49 MHz for cochlear implants) [68].

The near-field technology has shown to be effective for short distances between the implant and receiver, offering high energy transmission efficiency at low frequencies. Far-field technology, which utilizes microwave energy, has the potential for miniaturization but faces challenges with operating frequency. High frequencies are required for efficient energy transmission but are absorbed by the human body, while low frequencies reduce sensor sensitivity. To address these challenges, the resonant inductive coupling method for magnetic field energy transmission is a promising option for wireless energy transmission systems.

The passive radio frequency identification (RFID) system has the advantage of being energy autonomous. Implementing wireless power sources in implantable devices offers the potential for an uninterrupted and reliable power supply, eliminating the need for battery replacement and subsequent surgical procedures. This addresses the inconvenience of repeated interventions and mitigates the risks associated with battery leakage and toxic component exposure. Furthermore, the absence of a battery allows for a reduction in the size of the implant, as there is no longer a requirement for biocompatible and impermeable packaging [69]. It consists of a processing unit, sensor, and digital tag device with an implanted antenna and chip with an identification code. The reader device can provide both power and information exchange with the RFID system through its electromagnetic field. This technology can be useful for marking prostheses, sutures, stents, or orthopedic fixation to track the patient's health status. The tags can also be embedded in prostheses to collect and transmit data, such as electroencephalograms [70]. However, the system's limitations include the requirement for power for reading the signal and the shallow installation depth (currently less than 70 mm), which makes it challenging to place the tag inside internal organs [71]. A key issue is reducing the size and weight of such systems, which also helps minimize toxicity and other negative effects on the body.

## 3 Health monitoring implants

**Fig. 2:**
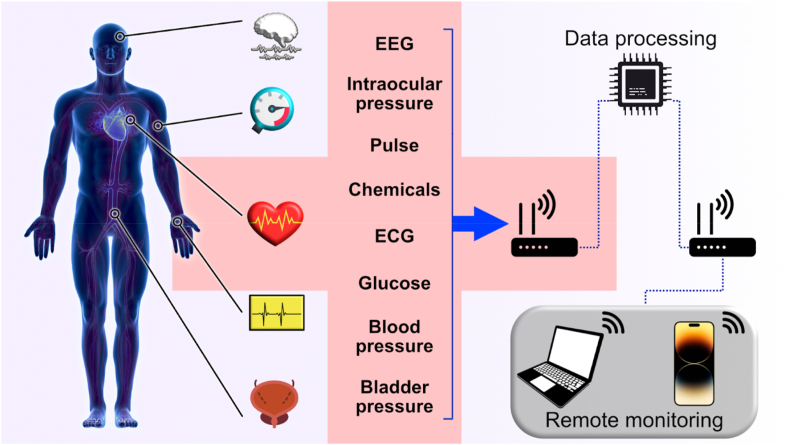
Monitoring structure using wireless medical sensor network.

The early detection and prevention of medical complications are of utmost importance, and monitoring implants could play a critical role in achieving this. These implants, which encompass antennas, sensors, and electrodes, are specifically designed to track essential parameters in real-time. Unlike traditional monitoring methods that require frequent visits to medical facilities, implantable monitoring devices enable continuous and remote monitoring of patients without the need for physical visits. This remote monitoring capability proves particularly advantageous during pandemics like COVID-19, as it reduces the risk of exposure to contagious diseases [58,72]. By utilizing implantable electronic devices for remote health monitoring, vital parameters can be monitored in real-time, facilitating the swift identification of potentially dangerous deviations and enabling the prompt detection of any issues with the treated body part or the implants themselves. Data are transmitted from the implanted device to an external one near the patient. This is accomplished through a wireless medical sensor network, as shown in Fig. 2 [73]. Implantable systems can record and monitor various biological parameters depending on the patient's health status, diagnosis, and type of implantable device. Commercial remote monitoring systems are tied to the manufacturer, meaning they are only compatible with devices produced by the same company [74]. The collected data are transmitted to the hospital database and the patient's physician regularly, with emergency notifications triggered in case of implant failure or a deterioration in the patient's health indicators. One example of the benefits of remote monitoring is a study where patients were monitored for one year. Using a multi-sensory algorithm, incorporating sensors for monitoring heart rate, heart sounds, chest resistance, breathing, and activity, increased the predictive sensitivity for preventing heart failure by up to 70% [75].

The advent of implantable electronic devices has revolutionized heart failure monitoring, enabling remote control and management. Cardiac-implanted electronic devices such as cardiac resynchronization therapy (CRT) with pacemaker function, CRT with defibrillator function, or implantable cardioverter-defibrillator can measure various parameters like thoracic impedance, heart rate variability, ventricular arrhythmias, and more. A study that followed 1650 heart failure patients found no difference in outcomes between active remote monitoring and traditional follow-up, although this may be due to the limited duration of the study [76]. Other studies have shown that remote monitoring can reduce hospital visits and healthcare resource use by up to 38% [77]. Using implantable hemodynamic technology to reduce pulmonary artery pressure has also decreased heart failure risk and hospitalization cases [78]. Hemodynamic devices track blood flow in the vessels by measuring differences in hydrostatic pressure, enabling the detection of a more significant deviation and number of diseases.

Recent technological advancements have led to the development of innovative devices that integrate monitoring functions with a range of diverse features. One notable example is the multifunctional hydrogel, which combines chemical cross-linking and stimuli-responsive interactions. This technological breakthrough enables comprehensive health monitoring and bidirectional neural interfaces, offering the potential for non-surgical disease diagnosis, treatment, and a transformative impact on healthcare [79].

### 3.1 Hemodynamic + pressure sensors

Pressure is a critical physical parameter that plays a significant role in the functioning of vital organs such as the brain, heart, nervous system, circulatory system, and kidneys [[80], [81], [82], [83]]. Strain gauges can also be used in orthopedic implants, such as knee implants, to study forces and torques, although the clinical benefits still need to be determined [37]. There are various pressure sensors, including piezoresistive, capacitive, fiber-optic, resonant, and piezoelectric [83]. These sensors typically consist of a deformable membrane element and a sealed cavity [84]. Meanwhile, microelectromechanical systems (MEMS) are used in membrane pressure sensors, which use capacitive [85] or piezo/tenso-resistive [86] effects to convert the membrane deflection into an electrical signal.

**Fig. 3:**
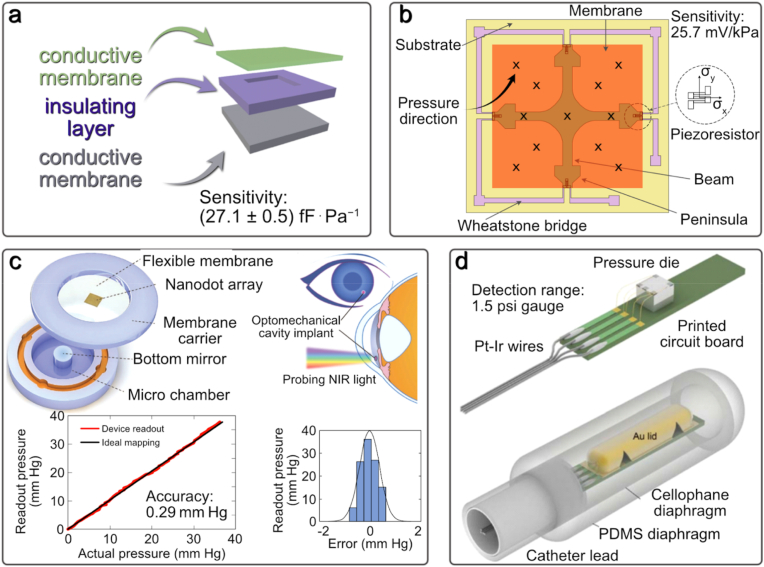
a) Diagram of sensor components of the touch-mode capacitive pressure sensor device (adapted from Ref. [87] under Creative Commons Attribution (CC BY) license). b) The structure of the combined cross-beam membrane and peninsula of a novel piezoresistive pressure sensor (Redrawn from Ref. [88]). c) Three-dimensional (3D) illustration of a micro-scale intraocular pressure sensor with nanotouch amplification and the principles of its operation, its location in the anterior chamber and experimentally determined spectra from the sensor in comparison with theoretically predicted spectra showing a high correspondence between sensor measurements (vertical axis) and digital pressure gauge readings (horizontal axis) (adapted from Refs. [88,89] under a Creative Commons Attribution 4.0 International License). d) The structure of the catheter tip with an integrated pressure sensor and electronics for measuring bladder pressure (Adapted by permission from Springer: Nature Science [90] © 2008).

Capacitive sensors have electrodes on the upper and lower surfaces of the sealed cavity. They can track minor deviations to measure ventricular pressure with a dynamic range of 5–300 mmHg and a sensor size of 2 × 2 cm^2^ [87]. Piezoresistive sensors use a strain gauge mounted on the membrane or have the membrane itself as a strain gauge. These sensors can respond to a pressure range of 0–50 mmHg with a full sensor size of 8 × 8 mm^2^ and be used to measure intracranial pressure [84,84]. Fig. 3a–b shows examples of capacitive [87] and piezoresistive [88] sensors, respectively. The typical size of a capacitive sensor is 3.5×15×2 mm^3^ [84], while the implanted piezo/tensoresistive sensor dimensions are around 2×2×1 mm^3^ or smaller. The capacitive sensor sensitivity is (27.1 ± 0.5) fF⋅Pa^−1^ within a pressure range of (0.5–8.5) kPa, while piezoelectric sensors can have a sensitivity of 25.7 mV/kPa for a pressure range of 0–5 kPa.

Pressure sensing technology has continued to evolve with new developments in biodegradable devices. One such alternative is a fiber sensor based on gas microbubbles, which embeds a hollow glass microbubble in a single-mode fiber to form a Fabry-Perot fiber interferometer. By measuring the reflected interference spectrum, this pressure sensor can achieve a sensitivity of 164.56 p.m./kPa [91]. Another development is a distributed fiber-optic pressure sensor based on Bourdon tubes that uses Rayleigh backscattering measured by optical frequency-domain reflectometry (OFDR). By tracking the local spectral OFDR system shifts, it is possible to determine the pressure applied to the Bourdon tube [92]. Another example is a sensor based on SiO_2_ with a sensitivity of ±1.5% and baseline changes of ±2.5 mmHg. Remarkably, the bioresorbable capacitive sensor can maintain its functionality for up to 25 days, showcasing its long-term monitoring capabilities [93].

Implantable sensors provide the means to monitor various hemodynamic parameters, including the blood flow rate. This particular parameter serves as a crucial indicator for assessing the condition of blood vessels and valves, as well as detecting arterial and venous thrombosis. Additionally, it aids in the diagnosis and monitoring of aneurysm treatments. Different types of sensors are available, such as magnetic, thermal, and capacitive.

Magnetic sensors operate on the principle of Faraday's law, converting the blood velocity into an electrical signal that accurately represents the flow velocity. Vennemann et al. demonstrated the effectiveness of an implantable wireless magnetic flow sensor with a diameter of 26 mm. This sensor can measure peak flow rates ranging from −24.5 L/min to 24.5 L/min, covering a wide range of typical flow rates in the ascending aorta. It exhibits a sensitivity of 0.070 L/min and can be placed at a maximum depth of 3 cm to facilitate wireless data transmission [94].

Thermal sensors, on the other hand, measure thermal energy transfer between two points along the flow. Lu et al. presented a thermal sensor comprising a surface-mount resistive heater and four negative temperature coefficient thermistors. The probe has cross-sectional dimensions of 2 mm width × 1 mm thickness. The reported sensitivity of this sensor is 1.2 ± 1.2 and 0.8 ± 0.8 mL/100 mL for artery and vein flow velocities, respectively. The measurement error is primarily attributed to uncertainties in blood percentages in muscles, and the temperature probe increase remains below 4 °C [95].

Capacitive sensors, on the other hand, rely on changes in capacitance caused by the bending of the dielectric layer when blood flows. Herbert et al. demonstrated the application of a capacitive sensor consisting of silver nanoparticle films on a soft elastomeric substrate. This wireless sensor enables the detection of biomimetic cerebral aneurysm hemodynamics with a maximum readout distance of 6 cm [96].

In addition to blood pressure, other parameters must be monitored for various medical applications. For example, monitoring intraocular pressure is crucial for early diagnosis of glaucoma. However, in practice, this parameter is rarely measured. A recent study demonstrated the development of microscale implantable sensors for continuous *in vivo* intraocular pressure monitoring. The study was conducted on rabbits and found that the optomechanical sensors, mounted on intraocular lenses or silicone haptics, could monitor pressure with an accuracy of 0.29 mmHg over the 0–40 mmHg range for 4.5 months. Results showed high compliance with theoretical data (Fig. 3c) [89].

Li et al. devised a remarkable solution for detecting abnormal respiratory events—a bioresorbable pressure sensor built upon a triboelectric nanogenerator. This innovative device exhibits outstanding sensitivity (22.61 mV/mmHg), exceptional linearity (R^2^ = 0.99), and remarkable durability (850 000 cycles). It has a lifespan of 5 days and undergoes complete degradation after 21 days, offering a temporary monitoring solution for respiratory conditions [97].

An implantable wireless pressure sensor system was developed to monitor the pressure in the bladder *in vivo* for urination monitoring. The system consists of a commercial pressure matrix in a catheter tip, amplifying electronics, a microcontroller, a wireless transmitter, a battery, and a computer to receive wireless data. The implanted device can continuously work *in vivo* on pigs for more than three days and transmit pressure data once per second with a resolution of 0.02 psi and a detection range of 1.5 psi gauge. This system can also be adapted for other organ pressure measurements and vital sign monitoring. Fig. 3d shows the sensor structure and its appearance after packaging [90].

The choice of pressure sensor depends on its intended implantation location. Compact capacitive sensors are suitable for implantation in almost any part of the human body, such as in an artery stent or the brain to monitor intracranial pressure. Additionally, capacitive sensors can measure pressures over a more extensive range than other sensor types, broadening their application scope. Integrating pressure and hemodynamic sensors into implants holds immense potential, considering the critical role of pressure in various organs. For instance, incorporating such sensors into vascular grafts or stents can provide valuable insights into thrombosis, a common post-installation complication. However, ensuring that the sensor does not contribute to occlusion is crucial, which necessitates designing a safe sensor that conforms to the shape and mechanical properties of the grafts or stents. Similar considerations apply to monitoring pressure in the brain, kidney, or intraocular regions. While polymer- or MEMS-based cavities can achieve miniature sizes, incorporating signal transmission and device power supply or generator components significantly impacts the overall size, possibly rendering it impractical for real-life applications.

### 3.2 Chemical sensing

Chemical monitoring in the human body is vital for detecting health issues and preventing disease progression. For instance, glucose plays a crucial role in metabolic processes, while enzymes stimulate brain activity and restore organs and tissues [98,99]. Dopamine and cortisol regulate mood, activity and are associated with various diseases. Measuring chemical deviations in the body can reveal various diseases, including latent ones, leading to improved patient well-being and disease prevention [100,101]. Moreover, the concentrations of all these compounds fluctuate greatly throughout the day, requiring their monitoring for reliable data collection or interventions.

Glucose is the most monitored compound due to its implications in diabetes and its consequences. Traditional blood tests under fasting conditions may not always indicate abnormalities, and approximately 50% of people with diabetes are unaware of their condition [102,103]. This highlights the importance of continuously monitoring glucose levels in the blood. A groundbreaking development in this field is the implantable potentiostat radiotelemetric system for glucose determination *in vivo*, which was introduced in 1994 [104].

Typical chemical sensors consist of a shell with an indicator or tag inside the core electronics and optical system [105]. Chemical sensors function on the basis of chemical reactions and physical phenomena. Based on their response nature, chemical sensors are classified as electrochemical, electrical, magnetic, thermometric, or optical. Among these, electrochemical sensors feature high sensitivity, fast response, design simplicity, and low cost [106], making them the ideal choice for real-time glucose monitoring. In electrochemical sensors, an electrical signal is generated by enzymes in response to chemical elements of the surrounding tissue or by a nanocatalyst. The intensity of the electrical current is proportional to the concentration of the analyzed component [107].

**Fig. 4:**
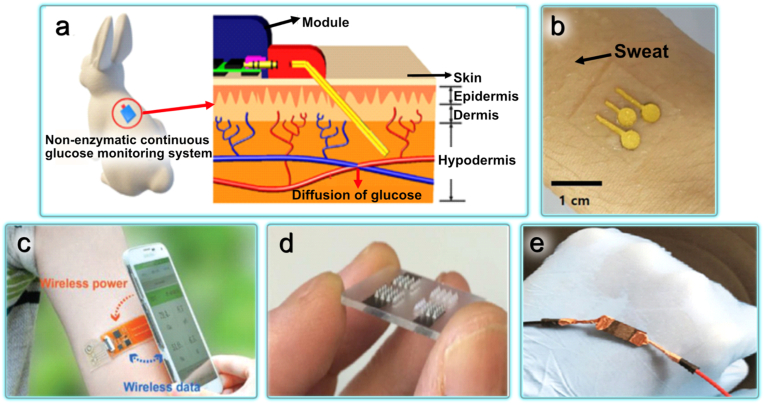
Modern glucose and electrochemical substance level studying methods. External skin devices: a) The skin-attachable, stretchable electrochemical sweat sensor for glucose and pH detection attached to the skin wet with sweat (Adapted with permission from Ref. [110] © 2022 American Chemical Society). b) Implantation of a robust, wearable, and non-enzymatic system for continuous glucose level monitoring in a rabbit and a cross-sectional non-enzymatic glucose sensor view (Adapted from Ref. [108] © 2022 with permission from Elsevier). c) Battery-free, wireless, and epidermal electrochemical system for in situ sweat sensing bonded to a subject's arm, with a smartphone for wireless power and data transmission (Adapted with permission from Ref. [111] under Creative Commons Attribution (CC BY) License). d) Microneedle sensor arrays for continuous glucose monitoring (Adapted from Ref. [112] with permission from the Royal Society of Chemistry). e) Self-powered implantable skin-like glucometer for real-time blood glucose level detection (Adapted from Refs. [108,113] under Creative Commons Attribution 4.0 International License).

Yoon et al. developed a wearable, reliable, and biocompatible system for continuous glucose monitoring that uses flexible stainless steel for improved adhesion between the polymer substrate and platinum (Fig. 4a). This system measured glucose levels in rabbits' interstitial fluid with 83% accuracy compared to blood glucose values [108]. To mitigate the problem of biofouling, Jayakumar et al. developed zwitterionic polymer biocoatings for glucose sensors. These coatings effectively reduce cell adhesion and enhance sensor sensitivity by approximately 1.5 times [109].

However, there are some issues with implantable glucose sensors such as painful insertion and infections and inflammation of fingertip [108]. To address this issue, Oh et al. demonstrated a wereable and stretchable electrochemical sensor for the glucose detection in sweat with high sensitivity of 10.89 μA mM^−1^ cm^−2^ (Fig. 4b) [110]. Using a smartphone, an ultra-sensitive optical converter was also developed for wireless glucose level monitoring (Fig. 4c) [114]. This optical converter includes oxygen-sensitive polymer points (Pdots) with glucose oxidase that sensitively and selectively detect glucose. Subcutaneous glucose levels can be tracked and evaluated through a smartphone application, utilizing optical images captured by the phone's camera. This innovative approach allows for convenient and real-time monitoring of glucose levels using a widely accessible device.

Implantable continuous glucose monitoring devices can aid in the self-management of diabetes, but their high cost and limitations in accuracy limit their usage. An alternative is a minimally invasive solid microneedle sensor array for continuous glucose monitoring (Fig. 4d). The microneedles penetrate the skin and come in contact with intercellular fluid, serving as glucose biosensors that monitor changes in concentration in real time. This technology has been tested on healthy individuals and patients with type 1 diabetes [112].

Patients who undergo prolonged glucose monitoring may develop skin allergies to isobornylacrylate (IBOA), commonly used in medical plastics and adhesives. To overcome this issue, the CGM Eversense system (Fig. 4e) offers an IBOA-free alternative, with its sensor, transmitter, and adhesives free from IBOA [114,115]. Currently, the Eversense system is the only commercial option for long-term glucose monitoring. However, there are limitations associated with implantable sensors, including the risk of inflammation, toxin release, and mechanical mismatch between the soft tissues surrounding the implant and the hard surfaces of the sensors. These limitations, combined with the challenges posed by biofouling, fibrous capsule formation, and inflammation, limit the viability of long-term monitoring systems [116]. Patients must periodically visit a clinic for device review to prevent these complications. However, this approach has limitations as the visits are infrequent and do not provide a complete picture of the patient's condition [117].

Tumor biomarkers play a crucial role in the diagnosis, prognosis, treatment, and control of specific types of tumors. In radiation therapy for cancer treatment, the presence of hypoxia in cancerous tissues indicates the effectiveness of the treatment, but non-invasive detection of oxygen deficiency poses challenges. To address this, Marland et al. developed an implantable sensor composed of a three-electrode electrochemical cell microfabricated on a silicon substrate. The sensor demonstrated a linear response to oxygen, with a sensitivity of −0.595 ± 0.009 nA/kPa, as shown in a sheep lung cancer model. However, the sensor's lifetime was limited due to biofouling, ranging from hours to days [118,119].

Dopamine, another important biomarker, can be monitored using smart implants. Dysregulation and deficiency of dopamine are associated with various pathological conditions, including mood disorders, Parkinson's disease, and other motion-related diseases [120]. Ali et al. presented a sensor comprising micro- and mesoscale structures coated with rGO (reduced graphene oxide) nanoflakes fabricated through Aerosol Jet nanoparticle 3D printing. This sensor enables rapid detection (60 s) with femtomolar concentration sensitivity. It has demonstrated the capability to sense dopamine in spiked human plasma and serum samples for up to 12 days *in vitro* [121].

While wearable sensors have been proposed for cortisol monitoring, implantable sensors for cortisol detection have not been realized except for electrophysiological signal recording through the adrenal gland [122].

Monitoring glutamate levels can provide valuable insights into the state of neurons. Excessive glutamate, for example, can lead to neuronal degeneration in spinal cord injury cases. Nguyen et al. have developed an amperometric biosensor utilizing platinum nanoparticles, multi-walled carbon nanotubes, and a conductive polymer on a flexible substrate. This sensor demonstrated excellent stability over 7 weeks in 0.01 M PBS, exhibiting a linear response and a sensitivity of 2.60 ± 0.15 nA μM^−1^ mm^−2^ at 0.5 V vs. Ag/AgCl. However, further investigation is required to assess issues related to biofouling and foreign body reactions [123].

The integration of chemical sensors into implants could prove beneficial across various applications. Monitoring dopamine or glutamate levels in neural interfaces or tumor biomarkers in cancer patients through breast or other implants could provide valuable information for assessing cancer treatment success following surgery. However, it is important to note that the maximum monitoring duration achieved for chemical signals currently ranges from a few weeks to a few months, limiting their usefulness to the initial stages. Therefore, the use of biodegradable materials for these sensors holds significant promise.

### 3.3 Temperature sensors

During active functions, such as electrical stimulation, implants can generate heat that may harm the body [124]. Similarly, in the case of orthopedic implants, temperature sensors can be utilized to monitor inflammatory reactions, where the temperature of the surrounding tissues may rise [125]. Consequently, there is a need for temperature monitoring in implantable devices.

Temperature sensors operate based on the resistance dependence of certain materials on temperature, such as resistance temperature detectors (RTDs) or thermistors. Thermistors are highly sensitive but nonlinear, while RTDs are relatively insensitive but exhibit a high degree of linearity. Park et al. introduced a biocompatible resistive-type temperature sensor utilizing a thermoresponsive hydrogel with a sensitivity of −0.0289 °C^−1^ [126]. Kumar et al. presented a flexible and biocompatible polymeric nanocomposite with a detection resolution of 0.5 °C within 30–40 °C range [127]. However, resistive sensors have drawbacks, including higher power consumption, self-heating, and low resolution [128].

Alternatively, capacitive sensors offer several advantages, such as high sensitivity, fast response time, high resolution, and lower power requirements. These sensors operate based on the temperature-dependent dielectric constant of the material. Lu et al. demonstrated a bioresorbable polyethylene glycol sensor with water barrier layers, exhibiting accurate operation for up to 4 days in rats, with an accuracy of approximately 0.5 °C and a precision of less than 0.05 °C [43].

### 3.4 pH sensors

Fluctuations in pH levels can provide valuable insights into various physiological, biological, and medical processes, such as enzymatic reactions, tumor progression, and wound healing. Real-time monitoring of pH levels in body fluids like sweat, tears, urine, and saliva can facilitate the timely detection of different diseases. Implantable pH sensors are crucial in tracking tissue acidity levels, such as cancerous tumors, or identifying inflammatory or infectious reactions following implantation [129].

The operation principle of pH meters is based on the potential difference created between the sensor element and the species in the body's environment. Cao et al. introduced an IrOx pH sensor with an Ag/AgCl reference electrode for monitoring pH levels in the pig's esophagus. This sensor demonstrated a sensitivity ranging from −51.1 to −51.7 mV/pH, high repeatability, and low hysteresis in the pH range of 1.9–12 at 25 °C. However, the challenge of regular surface cleaning needs to be addressed for practical applications of such a sensor [130]. Corsi et al. reported a bioresorbable nanostructured pH sensor capable of continuous monitoring for over 100 h in the pH range of 4–7.5, exhibiting a sensitivity of −6.2 ± 1 mV/pH *in vitro*. *In vivo* studies demonstrated stable local pH monitoring through the skin in mouse models for 30 min [131]. Another study by González-Fernández et al. introduced a pH sensor based on a tri-branched methylene blue redox system. The sensor exhibited stable operation *in vivo* on a sheep lung cancer model for 80 min, with a sensitivity of −56 ± 2 mV/pH in the pH range of 4.6–7.9 [132].

While significant progress has been made in improving pH-sensing systems, stability remains challenging. Electrodes may experience potential drift over time, making it difficult to obtain consistent measurements. Repeatability is another significant challenge for pH sensors, as it refers to their ability to produce consistent results when exposed to similar solutions. Achieving perfect repeatability is nearly impossible, but minimizing deviations is crucial for reliable pH measurements [133]. Additionally, biofouling poses a challenge in pH sensor development as it reduces the device's lifespan. To overcome this, antibiofouling coatings or materials with such properties are necessary.

Both temperature and pH shifts are common non-specific signs of inflammation and other critical issues like tumors. Inflammation often arises from infection or allergies, which are common complications after implantation surgery. Early detection of these problems is a crucial feature that can provide clinical benefits by enabling timely interventions and potential replacement of the implant itself.

### 3.5 Challenges in implantable sensors

Wireless smart implants offer a multitude of benefits in healthcare, including constant monitoring, post-operative rehabilitation, and rapid emergency response [134]. However, the long-term use of these sensors faces some persistent challenges. One of the main problems is immunological reactions, where the sensitive components are contaminated with proteins and other substances over time, encapsulated in fibrous tissue due to foreign body response, leading to changes in the output signals or failure of the sensors. To address this, specialized packages that prevent protein adsorption may be used. Depending on the package material, the lifetime can be from several days (for bioresorbable materials) to several years (for glass and titanium) [135]. For pressure sensors, there are also technical issues such as sudden shifts or gradual baseline pressure drift during intracranial pressure monitoring also pose a challenge [136]. Electrostatic charge can cause baseline displacement, affecting patient management [84].

Despite the huge potential of chemical sensors, a major challenge in the field of chemical sensing is the integration of electrochemical sensors with various types of implants. Currently, alternative sensing methods, such as surface-enhanced Raman spectroscopy, are being utilized [137,138]. However, electrochemical sensors have the potential to be the optimal choice for creating smart implants with chemical sensors, as they possess high sensitivity, simple design, low cost, and fast response capabilities. These properties are crucial for the real-time monitoring of vital indicators in the human body.

## 4 Electrical stimulation and neural interfaces

Electrical stimulation (ES) and monitoring systems activate and track various body parts, such as the brain and spinal cord, auditory nerve, retina, heart, etc. They have the potential to improve the quality of life for patients suffering from organ damage or paralysis, relieve chronic pain, and aid in the treatment of mental disorders. ES uses pulsed currents to restore the normal function of nerves and muscles affected by injury or illness. Both invasive and non-invasive methods are used for stimulation and organ monitoring. Invasive procedures, such as implantable systems, are considered more effective for electrical stimulation and nerve tissue, muscle, and organ monitoring.

The use of stimulating implantable systems is driven by the need for direct electrical stimulation for disease treatment when drug therapy is insufficient. They can provide an electrostimulation effect, allowing for the restoration of lost functions [139].

Neural interfaces (NI) are information exchange systems that connect the brain's electrical activity with an external device. Neurostimulation implants, such as deep brain stimulation [140], cochlear implants [140,141], nerve stimulation [142], visual implants [143], and cardiac stimulation [144], enable the restoration of lost functions through electrical stimulation.

### 4.1 Recording and stimulation mechanism and technologies

The principle of ES is based on applying electrical impulses to activate cells that respond to electrical signals, such as nerve, muscle, and glandular cells. This activation process results from a change in the membrane permeability to Na^+^, K^+^, Ca_2_^+^, and Cl^–^ ions. Upon applying an electric pulse, potential-dependent Na^+^ channels open, leading to an influx of Na^+^ ions into the cell along the concentration gradient. This results in membrane depolarization and the establishment of an action potential (AP), which triggers specific reactions in excitable tissue, such as substance secretion for glandular cells, the contraction for muscle cells, and nerve impulse conduction for nerve fibers.

**Fig. 5:**
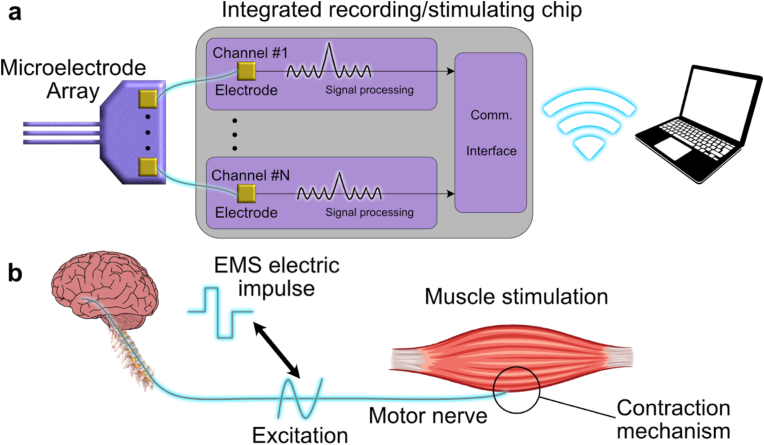
a) System architecture of the electronics part of a bidirectional NI (Adapted from Ref. [147] © 2022 with permission from Elsevier). b) The mechanism of muscle excitation.

NI utilizes electrical impulses to activate or detect action potentials, as shown in Fig. 5a. These interfaces can be either wired or wireless and consist of three key components: a tissue interface, typically electrodes that can be invasive or non-invasive; a recording and stimulation device for capturing neural signals and delivering stimulation; and a neural signal processing unit [145]. The system's location within the body depends on the device type and the intended stimulation purpose [146]. Fig. 5b depicts the muscle stimulation and recording mechanism. During stimulation, electrical impulses are applied to the motor nerve or directly to the muscle through the electrodes, exciting the cells and causing the muscle to contract. The contraction is then detected in reverse order: the muscle contracts, sending an impulse along the motor nerve, and the electrodes detect the electrical signal.

Additionally, recent developments in neurostimulation technology include the creation of an optoelectronic probe. This probe stimulates genetically photosensitized neurons with light and can record electrical signals. Incorporating electronic circuits into the optogenetic device enables the development of smart sensors for long-term or permanent implantation [148]. Moreover, flexible hydrogel sheets were created for syringe injection for optoelectronic and biochemical stimulation. The study showed that optoelectrical stimulation for two days contributed to an increase in neurite by 36.3% [149].

These advancements in neurostimulation allow for additional functions beyond stimulation, such as monitoring nerve tissue through transparent graphene electrode arrays. This transparency is crucial for monitoring the condition of the tissue during treatment and avoiding potential complications [150].

Due to the potential complexities posed by invasive NI, efforts are being made to develop non-invasive methods for stimulation. Commonly used non-invasive methods include Transcranial Electrical Stimulation (tES) [151] and Transcranial Magnetic Stimulation [152]. However, these methods generate a broad electric field that can't be precisely directed to a specific area in the nerve tissue.

ES is used in treating various diseases, but cardiovascular ones occupy a special place, as discussed in the following section.

### 4.2 Organ stimulation systems

**Fig. 6:**
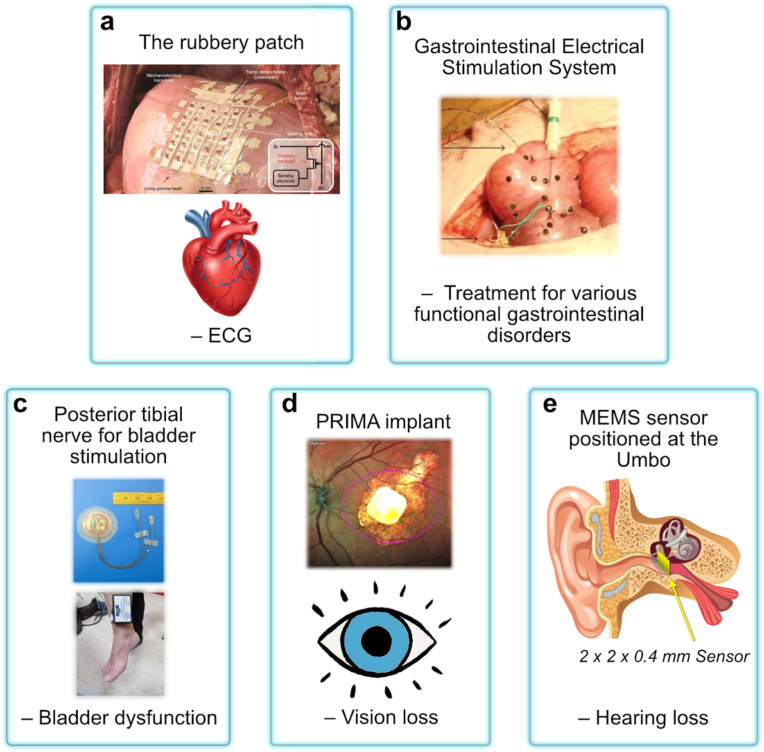
Frontier examples of current peripheral nerve stimulation/organ modulation and recording techniques with an example for a relevant disorder/application denoted underneath each technique. a) The rubbery patch on the epicardial surface of a living porcine heart. Inset: the circuit diagram for a single sensing node in the 5×5 active matrix (adapted from Nature: Electronics [154] © 2022). b) Gastrointestinal electrical stimulation system (adapted from Ref. [155] under Creative Commons CC-BY License). c) Posterior tibial nerve for bladder stimulation (Adapted from Ref. [156] under The Creative Commons CC-BY License). d) Fundus photo of a patient with the PRIMA implant inside the geographic atrophy area. The magenta oval illustrates the size of the beam (5.3 × 4.3 mm) projected onto the retina (adapted from Ref. [157] under Creative Commons CC BY license). e) Representation of a 2 × 2 × 0.4 mm^3^ MEMS sensor positioned at the Umbo [158].

Cardiovascular diseases are still the leading cause of death and disability globally. Pacemakers are commonly used to regulate the functions of cardiac (Fig. 6a) and gastrointestinal muscles (Fig. 6b). They have become more practical to implant with the invention of transistors and the miniaturization of generators and electronics. However, the main challenge is to extend battery life so that surgery for battery replacement is no longer necessary [153].

Treatment of gastrointestinal dysfunction using ES has been successful in restoring motility, particularly post-surgery. A study [159] of 9 patients with irritable bowel syndrome showed symptom improvement after six months of ES treatment. The pacemakers were implanted in a subcutaneous pocket in the inguinal area, producing positive results in all patients.

Additionally, pacemakers were implanted into the stomach walls to suppress appetite and treat obesity. The study [160] comprised 11 patients and showed an average weight loss of 10.4 kg in six months. The exact mechanism behind the increased satiety feeling is not yet understood. Still, it is thought to be related to decreased appetite-affecting hormones or changes in gastrointestinal tract peristalsis.

Electromyostimulation is a standard method of muscle stimulation. It is used to aid in muscle recovery post-injury or surgery. Non-invasive methods are the most popular approach because invasive techniques are accompanied by painful sensations and do not allow long-term data collection [161]. However, invasive techniques offer more precise stimulation of specific muscles. When stimulating a muscle directly, it contracts following a single stimulus. This process comprises three phases: the latent period (time between the stimulus and the response), the contraction phase, and the relaxation phase.

It is also helpful to restore organ function with nerve stimulation. For example Janssen et al. showed successful treatment of refractory overactive bladder syndrome with electromagnetic stimulation of the posterior tibial nerve through the monopolar platinum electrodes (Fig. 6c) [156]. Elefteriades et al. [162] demonstrated the effectiveness of monopolar platinum band electrodes implanted in 12 patients with complete respiratory paralysis for diaphragm stimulation. All patients were successfully conditioned and achieved permanent ventilation. Smart implants can also be used to restore sensory organ functions, such as vision [163] and hearing [164,158] (Fig. 6d–e). Electrical impulse stimulation can also address chronic pain syndromes by suppressing aches and pains that cannot be addressed through other means.

### 4.3 Pain management

More than a third of the United States and Europe population suffer from persistent chronic pain, which can negatively impact the quality of life [165]. Standard pharmaceutical methods for relieving pain are not always effective, can be expensive, and have potential side effects. Neurostimulation (NS) is an alternative treatment option, which includes spinal cord stimulation (SCS), peripheral nerve stimulation (PNS), peripheral nerve field stimulation (PNFS), and transcutaneous electrical nerve stimulation (TENS) [165].

Neurostimulation therapy offers several advantages over standard pharmaceutical methods, including potentially lower costs and milder side effects. While the cost-effectiveness of neurostimulation therapy compared to pharmaceutical approaches may vary depending on the specific condition and patient characteristics, it has the potential for long-term cost savings. Although there may be higher initial costs associated with implanting devices or electrodes used in neurostimulation therapy, the sustained pain relief it provides can reduce the need for ongoing medication and associated healthcare visits [166,167].

In contrast to standard pharmaceutical methods, neurostimulation therapy has fewer systemic side effects since it primarily targets the nervous system instead of affecting the entire body. While localized side effects such as discomfort or irritation at the implantation site may occur, they are generally milder and more confined to the specific area [168].

Multiple studies have provided compelling evidence of the efficacy of neurostimulation therapy in effectively managing a wide range of pain conditions. These include chronic neuropathic pain, acute postoperative pain, postamputation pain, and low back pain. The collective body of research supports the effectiveness of neurostimulation therapy as a valuable treatment option for these diverse pain conditions. For instance, Biurrun Manresa et al. [169] used surface ES with silver chloride electrodes to reduce pain intensity in 17 patients, while Mainkar et al. [170] used temporary, percutaneous PNS to reduce pain in 7 out of 12 patients. Additionally, Skaribas et al. [171] reported successful complex regional pain syndrome treatment using electrodes implanted into the spinal cord.

### 4.4 Mental disorders

**Fig. 7:**
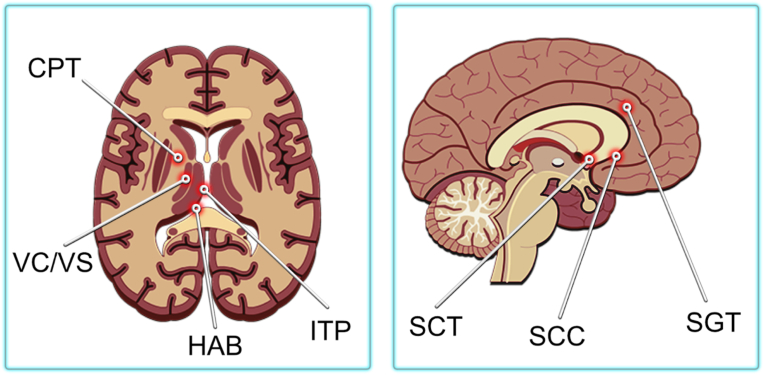
Location of various targets for ablation or DBS for psychiatric disorders. CGT - cingulotomy, CPT - capsulotomy, HAB - habenula, ITP - inferior thalamic peduncle, SCC - subcallosal cingulate, SC - subcaudate tractotomy, VC/VS - ventral capsule/ventral striatum.

Neuromodulation is an effective treatment option for mental disorders involving abnormal thoughts, emotions, behaviors, and changes in physical functioning. One of the treatment methods is deep brain stimulation (DBS) with ES. Depending on the illness, DBS can stimulate different brain parts (Fig. 7). Kahan et al. [172] studied the mechanisms and efficacy of DBS for Parkinson's disease treatment. Eleven patients with Parkinson's were treated with chronic DBS, and all experienced some degree of clinical improvement. In particular, several experienced a reduction in tremors when the DBS was activated. In a comprehensive 12-month study conducted by Zhang et al., the efficacy of deep brain stimulation (DBS) was assessed in a cohort of 85 patients with Parkinson's disease. The results revealed a statistically significant mean improvement in PDQ-8 and UPDRS III scores, indicating the positive impact of DBS as a treatment intervention for Parkinson's disease.[ [173,174] Furthermore, Sankar et al. [175] investigated the effects of DBS on the treatment of six patients with Alzheimer's disease.

NS has been demonstrated to reduce brain atrophy associated with neurodegenerative diseases, such as Alzheimer's, and reduce aggression in mentally disabled patients. For example, after a year of stimulation, patients with Alzheimer's significantly increased their hippocampus size. Additionally, NS has been used to treat obsessive-compulsive disorder, with Nuttin et al. [176] and Greenberg et al. [177] showing successful results in their studies.

In Nuttin's study, deep brain stimulation at 100 Hz and 210 ms pulse width resulted in a marked decrease in aggressive behavior in 9 out of 12 patients. Similarly, Greenberg's study found that more than 60% of the 26 patients experienced a clinically significant reduction in symptoms and functional improvements after 3–36 months of DBS.The implantation of electrodes for DBS poses several challenges, including brain hemorrhage and adverse effects from incorrect parameter selection [178]. An alternative treatment for neurological and psychological disorders is repetitive transcranial magnetic stimulation; this non-invasive method effectively treats refractory depression.

### 4.5 Stimulating cell growth

ES can be used to promote the growth of neural cells, although the exact mechanism of this effect is not yet fully understood. One hypothesis suggests that Schwann cells, a type of glial cell, are actively involved in the regeneration and growth of nerve cells. These cells produce a protein known as neuron growth factor (NGF), which is responsible for the growth and development of neural cells, particularly axon growth [179,180]. *In vitro* studies by Huang et al. [179] have shown that ES can result in a fourfold increase in NGF production from Schwann cells. Similarly, Song et al. [181] observed the effectiveness of ES in restoring nerve fibers, as electrical co-stimulation led to a four-fold increase in the release of brain-derived neurotrophic factor, which supports and encourages the growth of neurons. Li et al. reported an implantable battery as the power source for in situ electrical stimulation and showed remarkable regeneration of the injured long-segmentsciatic nerve of rats [173]. Cheng et al. [182] demonstrated that a combination of cyclic strain and electrical co-stimulation could promote the differentiation of stem cells into neural cells, which had more branches and longer neurites than those that were only exposed to strain or ES. Zhu et al. provide a comprehensive summary of the stimulating impact of electrical and electromagnetic fields on neural stem cells. They propose a potential mechanism where electrical stimulation triggers the reorganization of cytoskeletal filaments and activates various pathways, receptors, and proteins. These molecular responses are responsible for promoting cell proliferation, enhancing cell survival, and facilitating cell mobility [183].

ES can promote not only the growth of nerve cells but also has a positive impact on bone tissue. It is thought that ES triggers the release of transforming growth factor-beta through a calcium/calmodulin pathway, which is associated with cell growth and differentiation [184]. Fonseca et al. studied the effects of ES on bone regeneration and found that it positively influenced osteogenesis treatment [185]. Wang et al. made a 3D biomimetic optoelectronic scaffold and showed an improvement in bone regeneration in rat models with increased mineral density and volume of bone trabeculae [186].

The success of electrical stimulation in modulating nerve and muscle cell activity and promoting tissue growth holds promising clinical implications. This technology could potentially aid in restoring muscle function, managing pain, and expediting the recovery process. It is conceivable that smart implants integrated with such stimulating components might lead to enhanced recovery and reduced pain. Nevertheless, further extensive research is needed to fully explore and harness the potential benefits of this direction.

### 4.6 Challenges in electrical stimulation and neural interfaces

Since maintaining stable electrical contact with the surrounding tissues is necessary for ES and NI functioning, electrode encapsulation is not possible and NI material must not exhibit toxicity or cause allergy. Implantable NI can be divided into three categories: microwires, silicon microneedles, and planar metal arrays [187]. However, using standard materials greatly limits the possibilities, manipulations performed, and duration, negatively affecting the tissues adjacent to the implant. Materials such as silicon, thermoplastics, and elastomers, which limit service life, are too hard for implantation. Metal electrodes, such as stainless steel, tungsten, or platinum/iridium, cause tissue damage and inflammation. Avoiding the use of adhesives or sutures to contact NI to the nerves is also desirable and can be achieved by making extremely conformal ultra-thin conductors [188]. In addition, the body's reaction to the implant, which means the glial scar formation, leads to signal distortion and the inability to register the signal [189].

The resting membrane potential typically measures around −60 mV, whereas extracellularly recorded signals can reach several hundred microvolts. When recording signals from the peripheral nervous system, unwanted artifacts from muscle activity or movement can overlap with the desired signal. To mitigate this issue, one potential solution is to position the recording electrodes as close as possible to the specific tissue area of interest. Target tissues or individual nerves may be located at various depths, requiring electrode penetration into the tissue. However, this can pose a risk of nerve damage. Addressing this challenge involves selecting materials with suitable properties and designing electrodes of appropriate shape and size to minimize potential harm and optimize recording quality.

Neurostimulation systems usually perform stimulation in an open cycle mode. In this case, the therapy is programmed once and does not change depending on changes in the patient's symptoms or physiological parameters, such as heart rate, blood pressure, respiratory rate, *etc*. [190] It is proposed to use closed-loop systems to set up therapy when changing physiological parameters. This will ensure safer and more effective treatment and reduce side effects such as impaired speech, gait, and balance [191]. Feedback technology allows reprogramming neurostimulation in real-time without needing intervention by a doctor or a patient. The closed-loop module's main problem is stimulation artifacts that obscure any neural activity near the stimulation site for tens or hundreds of milliseconds. The stored electrode charge, which ultimately generates the artifact, introduces problems for long-term stimulation protocols, as it could cause ion migration or general recording system saturation. At the moment, these artifacts can only be eliminated by stopping recording for a short time. But this solution leads to information loss [192].

## 5 Materials and technologies

### 5.1 Substitute implant materials

The materials used for implants must be biocompatible, have suitable mechanical properties for the tissue they are replacing, and ideally, not require removal or be biodegradable [193]. Biodegradable materials must meet the following criteria: (1) it should possess mechanical properties that are compatible with the specific site of implantation. This ensures that the device can withstand the physiological conditions and forces acting on it without compromising its structural integrity. (2) it should not trigger an inflammatory response in the surrounding tissues. Inflammation can hinder the device's functionality and lead to complications. (3) the decomposition time of the implantable device should align with the optimal duration required for it to perform its intended function. This ensures that the device remains effective for the necessary duration without any adverse effects. (4) the materials should decompose into non-toxic and safe byproducts that can either be excreted from the body or remain inert within the body tissues. This is crucial to prevent any harm or adverse effects on the patient's well-being. (5) the materials should be cost-effective and undergo an efficient manufacturing process tailored to the specific application. By meeting these criteria, implant materials can enhance patient safety, promote successful integration, and potentially eliminate the need for additional surgical interventions. However, choosing a biomaterial that fits all medical needs is challenging and requires specific research for each application. For instance, bone implants, suture implants, and porous structures for tissue engineering differ greatly in their required physical and chemical properties [194]. Bone implants must be able to support a broken bone until it heals and then dissolve [195], be removable [196], or provide permanent support to the bones [197]. Therefore, they should be durable, ductile, and preferably radiopaque. In contrast, suture implants should be elastic and flexible. Biodegradable implants must be completely biodegradable into safe components and excreted from the body [198]. Porous bone-support implants must withstand anatomical loads to prevent injury, have a surface that promotes cell adhesion and growth, and have high porosity for cell ingrowth and proper vascularization [198,199]. There is also a size and weight constraint, as the device must weigh less than 2% of the patient's body weight [200]. Implants that are too heavy or large will put excessive pressure on the surrounding tissue already injured from the implantation procedure.

Recent research aims to develop implants that can fully dissolve in the body, eliminating the need for subsequent removal surgery [40,201,202]. However, in most cases, the implants should be bioresorbable, meaning that they gradually break down and are replaced by natural tissue over time. The biomaterial's decomposition time must align with the tissue regeneration and healing process for optimal recovery [203,204]. It is challenging to strike a balance between the implant's mechanical strength and gradual degradation.

The materials used for implants in direct contact with the human body can be classified into three categories: metals, ceramics, and polymers. Different implant types are selected based on the purpose, location, risks, and requirements of the implant. For example, for bone support, mechanical strength is crucial [205], for cardiac sensors, the chemical composition must prevent blood clot formation [206], and for neurostimulation implants, efficient pulse transmission is important [207]. Innovations in recent years have combined different material types, such as carbon-based materials and oxides, to create new implant options [[208], [209], [210], [211]]. However, the choice of an implant type should be specific to each case, considering factors such as biodegradability, lifespan, flexibility, and durability. Biodegradable implants dissolve in the body and eliminate the need for removal, but they have a limited lifespan [212]. Durable implants are used for bone support [213]. Flexible materials are suitable for sensors and placement in soft tissues [214]. Thus, an individual implant type selection is necessary for each case, depending on its installation purpose.

#### 5.1.1 Metals

Metals were originally used for bone implants to provide the necessary mechanical support, and they still dominate orthopedic devices today [[215], [216], [217]]. Later, metals were also used in non-bone tissues, such as arteries (coronary stent) [218]. The most popular and widely used metallic biomaterials for hard tissue implants include stainless steel [219], cobalt-chromium alloys [219,220], and titanium [221]. Additionally, materials with shape memory, such as NiTi (nitinol) [222], tantalum [223], zirconium alloys, and silver, are also used for these purposes. However, metal materials can cause allergic reactions in 10 – 15% of the population, leading to implant failure [224,225]. Despite being stronger than bones, metal bone implants have a limited service life of 20–25 years, with the majority of problems arising after 15 years due to the inability of artificial materials to recover from wear [226]. Noble metals like gold and platinum have been widely employed in the development of neural interface electrodes. This is attributed to their excellent electrical conductivity and ease of processing, particularly in high-density arrays. However, metal electrodes exhibit drawbacks such as high stiffness and low electrochemical capacity. As a result, there is ongoing active research focused on exploring new materials that can overcome these limitations and offer improved performance in neural interfaces [227].

#### 5.1.2 Biodegradable metal alloys

The limitations of traditional metal implants have led to research and development of biodegradable metal alloys such as magnesium, yttrium, strontium, iron, zinc, and metal ceramics [228]. These materials show good biocompatibility and offer sufficient support while safely decomposing in the body [203,204,204,229]. For example, magnesium alloys mixed with calcium, strontium, or zinc have been studied for their potential use as implants and for monitoring their degradation [[230], [231], [232], [233]]. The biodegradation properties vary based on the alloy components. Magnesium is a promising candidate for bone implants, with mechanical properties similar to human bones. A long-term clinical study of 53 cases showed that an Mg–Ca–Zn alloy implant could fully dissolve in the body and be replaced by new bone tissue [234]. However, the problem of such biodegradable metal alloys remains the release of metal ions, H_2_, and/or particles due to their corrosion, which causes systemic toxicity in humans [235].

#### 5.1.3 Bioactive coatings

Bioactive coatings enhance the properties of metal implants, making them more biocompatible and biodegradable [226,236]. One such example is bioactive glass, which is highly biocompatible and can integrate with human tissues to promote regeneration. Its degradation rate is similar to the rate of tissue repair [237,238]. Additionally, glass ceramics and glass polymer composites are used to coat metal implants to improve their mechanical strength, adhesive properties, and bioactivity [[239], [240], [241]]. These materials exhibit minimal adverse reactions, reducing the risk of inflammation and rejection, leading to improved patient outcomes [242]. Additionally, certain types of bioactive glasses stimulate bone tissue growth, enhancing the integration of the implant with the surrounding biological environment [243]. In addition, sometimes glass or ceramics are used not only as a coating, but also as the main material of the implant [239,240].

#### 5.1.4 Polymers

Polymers are versatile biomaterials and can potentially replace other materials used in implants, such as ceramics, metals, and alloys [194]. Polymers can be divided into three categories: natural, synthetic, and microbial biodegradable polymers [[244], [245], [246], [247]]. Biodegradable polymers, such as copolymers of polylactic acid (PLA), polycaprolactone (PCL), polylactic-glycolic acid (PLGA), polyglycolic acid (PGA), and poly (1,8-octanediol-*co*-citrate) (POC) are widely used in the creation of transient electronics and biodegradable implantable electronics [[248], [249], [250]]. Biodegradable implantable electronics based on polymers that decompose in the body have also been created [251,252].

Biodegradable polymers have unique properties, such as biodegradability and biocompatibility, making them valuable in biomedical applications. Moreover, biodegradable implants are generally known for their low risk to cause inflammatory reactions, if there is no need to remove them [253]. Numerous studies have demonstrated the absence of inflammatory reactions in biodegradable implants [254,255]. However, if it is necessary to extract a biodegradable implant for one reason or another, the risk of inflammatory reactions increases [256], although in recent times the need for the extraction of biodegradable implants is becoming less frequent [257,258]. Most studies of permanent non-biodegradable implants are accompanied by inflammatory reactions [259]. Chronic inflammation, characterized by long-lasting immune responses, is generally not observed with biodegradable implants [260]. It is important to note that the extent of the inflammatory response can vary depending on the specific material composition, the implant's interaction with the biological environment, and individual variations in immune responses. Manufacturers and researchers in the field of biodegradable materials strive to minimize the potential for inflammatory reactions by carefully selecting and developing materials that are less likely to provoke such responses. Therefore, the best solution would be to use materials with no or minimal inflammatory reactions, which are biodegradable materials.

To achieve optimal results, a combination of several biocompatible materials is often used instead of relying solely on one material. This allows for the creation of the best structure for a specific application [211,[261], [262], [263], [264]]. For example, a combination of biodegradable poly(2-hydroxyethyl methacrylate) (pHEMA) hydrogels and polymer polycaprolactone were used to develop engineered tissue structures. Polycaprolactone acted as a crosslinking agent and improved the properties of the pHEMA hydrogel [265]. These developments in polymers and materials have significant potential in the healthcare industry.

In the context of our review, these materials play a crucial role as carriers for sensing or stimulating components. Advancements in flexible electronics have made it relatively easy to create circuits on polymer substrates. However, working with metal or ceramic substrates presents unique challenges. Metals, despite requiring electrical insulation, can still cause interference with electromagnetic wave propagation, commonly used for signal or power transmission. On the other hand, ceramics, being insulators, make excellent substrates. However, ceramics used in implants are optimized for porosity to enhance cell adhesion and proliferation, making it difficult to achieve consistent and robust electronic components on such surfaces.

### 5.2 Electronic component materials

Creating smart implants that perform monitoring and stimulation functions and provide tissue support presents additional challenges. Unlike traditional electronics that must remain stable for long periods, biodegradable electronics are designed to dissolve safely and completely or partially over time [266,267]. There is also ongoing research to create "green" electronics using biodegradable materials to reduce environmental issues [268]. Biodegradable organic materials, including natural or synthetic polymers, are used as the passive components of implanted electronics, while metals and inorganic semiconductors are used for the active components. This allows creating "smart" implants with integrated electronic components, enhancing their functions. Another option is to encase well-developed, non-biocompatible electronic components in a biocompatible, impermeable shell, as was done for heart pacemakers. For example, Elon Musk's team used this approach for "Neuralink," an implanted NI in the brain, where all electronic components are housed in a titanium enclosure [269]. A study by Van Gaalen et al. [270] also developed a titanium implant for the hip joint to monitor the implant's condition, with a printed circuit board containing electronic components hidden inside. However, this approach limits the ability to control the device's mechanical properties and affects its weight and size while also preventing direct contact with tissues or liquids, which is often necessary for sensing or stimulating components.

#### 5.2.1 Semiconductors

Inorganic semiconductors such as mono-Si NMs (30–300 nm), polycrystalline silicon (poly-Si), amorphous silicon (a-Si), germanium (Ge), silicon-germanium alloy (SiGe), indium-gallium-zinc oxide (a-IGZO), and zinc oxide (ZnO) are widely used in the development of smart implants. The dissolution rate of these materials in saline solution depends on various factors, including the composition of the saline solution, doping levels, temperature, protein and ion types, deposition conditions, and film density [[271], [272], [273], [274]].

#### 5.2.2 Substrates and insulators

Polymer materials are commonly used as substrates and insulators in biomedical implants, with the main requirement being biocompatibility and appropriate mechanical properties. If used for device packaging, the material must have Young's modulus similar to surrounding tissue, high tensile strength, good flexibility, and complete impermeability [275]. Due to their low surface energy and surface shrinkage, silicones, such as perylene and polydimethylsiloxane (PDMS), are often used in biomedical applications [276,277]. Perylene C is used in long-term electronic device implantations [278], neural sensors [278], as a substrate for electrodes [278,279], and in cortical probes [280]. However, perylene has low mechanical strength and weak adhesion, which limits its use [275].

There is research exploring the use of fully biodegradable single-crystal silicon photovoltaic platforms for powering biomedical implants, with the platform completely decomposing in the body in 4 months [281]. Smart shape-memory polymers and hydrogels are alternative materials for standard substrate materials and provide greater biological compatibility due to their softness [[282], [283], [284]]. In a 2017 study, off-stoichiometry thiol-enes-epoxy (OSTE+) polymer samples showed a decrease in fluorescence intensity of activated microglia/macrophage biomarkers compared to silicon material, indicating a closer hardness to brain tissues and increased service life for NIs [285]. The possibility of high-quality clinical trials with long-term therapy and data recording is promising.

#### 5.2.3 Conductors

Biomedical implants require biocompatible, stable conductors and have properties similar to standard conductors. Two categories of materials are used for this purpose: organic and inorganic [286].

Inorganic conductors include metals like gold, iron, magnesium, zinc, molybdenum, and tungsten, which have high conductivity and energy density. However, most of these metals are bioresorbable, which means the body absorbs them, and the dissolution rate must be checked to prevent toxicity.

Organic conductors, such as conductive polymers like polypyrrole, poly(3,4-ethylenedioxythiophene), polyaniline, and their composites, have high flexibility and both electronic and ionic conductivity, which is necessary for biomedical engineering [287]. However, organic conductors have low cell affinity and are not osteoinductive, limiting their use in tissue engineering [287,288].

The demand for a flexible and conductive material for flexible electronics has led to the development and research of nanomaterials such as carbon nanotubes [289,290], metal nanowires [291], organic transparent films [292], and their composites. Also, reduced graphene oxide (RGO) and nano-hydroxyapatite (nHA) found their application in 3D porous biomimetic scaffold development for bone regeneration [293]. These carbon-based materials have great potential in biomedicine and implants, with improved physicomechanical properties and enhanced bioactivity. They can serve as electrode materials in various implantable devices due to their high surface area, good conductivity, flexibility, and biocompatibility [294,295].

While carbon nanotubes have excellent mechanical, thermal, electronic, and biological properties, there are concerns about their toxicity, biosafety, and biodegradation. Further clarification of these properties is necessary to use carbon-based materials in medicine in the near future [209].

### 5.3 Challenges in smart implant materials and technologies

Medical electronic implants have become a rapidly growing field, as they offer a promising solution to a range of clinical problems, such as monitoring vital body indicators, stimulating tissues and organs, and providing closed-loop health monitoring and therapy [296,297]. The use of MEMS manufacturing technologies, such as lithography, has enabled the production of bioresorbable active electronics. However, traditional fabrication techniques have limitations when working with biodegradable materials, as exposure to water and high temperatures can alter their properties [298].

To overcome these challenges, researchers are exploring alternative MEMS manufacturing techniques, such as injection molding, stamping, and stereolithography, which can be used to create MEMS devices using polymers [[299], [300], [301], [302], [303]]. For instance, metal injection molding of Mg–Ca alloys has been used to produce orthopedic implants with specific mechanical properties [304]. A flexible conductive elastomer PDMS (CPDMS) strain sensor was also created through stamping using PDMS and conductive carbon nanoparticles, simplifying the sensor creation process by reducing the number of machining steps [305]. Stereolithographic 3D printing has also been used to create cross-linked polyethylene glycol diacrylate hydrogels containing ibuprofen, offering a new approach to creating pharmaceutical hydrogels [306].

Alternative methods such as embossing, layering, and lamination are being used instead of strong solvents and chemicals commonly used in traditional MEMS fabrication to prevent degradation of biodegradable substrates [252,307]. Additionally, electronic layers are often first applied to a standard silicon substrate and then transferred to a biodegradable substrate [308].

Flexible electronics structure includes an encapsulating layer or substrate, multifunctional sensors for receiving signals from the body, circuits for processing the received signals, and power a source [309,310]. For encapsulation layers and flexible substrates, biodegradable materials are used, for example, polymers [311] or insulating silicones of medical grade [312], and various self-healing materials [312]. The next component is a flexible electronic circuit responsible for the electrical transmission between functional components and human-machine interfaces. There are several approaches to create a flexible circuit, such as liquid metals, modification of materials, and designing circuit geometry architecture [25]. Liquid metals, such as liquid-phase eutectic gallium indium (eGaIn), are usually injected into a closed elastomeric substrate, which allows them to function under deformations [313]. However, damage to the chain or some metal leakage poses a huge danger to the human body, but some still have low toxicity [313]. An alternative way is using biocompatible components based on metals. In this case, they are applied or printed on a flexible substrate using micro/nano production methods. Nanowires based on various materials, including metals, proved themselves well in this application [314,315]. But still, the material modification is usually difficult to reproduce and limited in deformations, so researchers are studying other ways to create flexible circuits [316]. The design method of the electrical circuit of the geometry architecture involves the creation of a wavy [316,317], serpentine [318], kirigami [319], or 3D architecture of a conductive structure that, when deformed, will not lead to the material destruction. The power source is used for stable and continuous bioelectronic operation. The power source in flexible electronics can be rechargeable batteries, a solar cell, piezo, tribo, thermoelectricity, or a biofuel cell. Although rechargeable batteries have a wireless charging function, they still require periodic charging. The main flexible electronic component is stretchable electrodes, which perform the main work-recording or monitoring any indicators. Flexible transparent electrodes have been widely studied recently and occupy a leading position in optoelectronics [320].

Biosensors play a crucial role in electronic medical implants as they can detect various types of physiological signals, both physical and biochemical. These sensors can be implanted or worn on the body, and new advancements have made it possible to detect biomarkers such as heavy metal ions [321], glucose [321], cortisol [322], and others [323]. Although there are limited technologies for creating biodegradable electronic devices, some have shown comparable results to traditional electronics [324,325].

## 6 Roadmap for smart implant development

Traditional medical monitoring approaches have limitations and place a burden on healthcare providers, leading to reduced quality of care due to high patient volumes and frequent in-person visits. Conventional methods such as X-rays and MRI often fail to provide a comprehensive understanding of an implant's condition. Complications and problems with implants are typically identified only when symptoms like pain or implant failure become apparent, highlighting the need for constant and high-quality implant monitoring.

Efforts are underway to develop new methods for implant monitoring, including the use of non-invasive visual sensors for real-time monitoring of implant dissolution rates. However, these methods may not be suitable for deep-seated implants, such as those in the thigh bone. The COVID-19 pandemic has further emphasized the issue of hospital-acquired infections and the shortage of qualified medical personnel, making remote monitoring an important solution to alleviate the strain on healthcare providers and improve the quality of care by reducing the need for frequent clinic visits.

While there is a continuous effort to improve implantable sensors and stimulating devices for better biocompatibility, performance, sensitivity, precision, and accuracy, the optimization of these technologies can be optimized by augmenting substitute implants with implantable electronics. By integrating such technologies, a single implantation procedure could provide additional benefits such as monitoring the implant and patient condition, detecting complications at early stages, and potentially enhancing recovery.

For various implants, such as those used in angioplasty or bypass procedures, in situ pressure monitors could be developed to timely detect thrombosis or occlusions, benefiting a large number of patients. However, challenges remain in terms of compact sensor design that does not promote blockage, power supply, and signal transmission. An elegant possible solution is creating an inductive stent powering two soft pressure sensors [326]. Using passive elements in conjunction with a reader system or systems powered by the body itself, rather than batteries, shows promise. Signal transmission for deep-seated implants is best achieved through radio frequencies and inductive coupling, although antenna size is limited by the frequencies used. Implementing several sensors into one implant powered by the same coil is a possible improvement that offers more information and a compact size [327]. Of course, biodegradable implants require consideration of their gradual decline in performance over time.

Implementing pH and temperature sensors in most implants could be a solution to monitor local inflammation caused by infection, tissue damage, or allergy, potentially improving recovery outcomes. Challenges arise when dealing with implants that experience significant mechanical loads, such as orthopedic or breast implants, as the electronic components must withstand these loads without failure. Additionally, the complex 3D shape of implants and the trend towards personalized implant shapes in modern medicine pose technological challenges for fabricating circuits with reproducible and robust electrical performance on complex surfaces.

Based on these considerations, several unique challenges for smart implants can be identified:

(1) **Integration of Robust Electronics:** Develop materials and technologies that enable the integration of durable electronic components into implant surfaces or bodies while maintaining mechanical properties, avoiding size increase, and minimizing the risk of failure.
(2) **Fabrication on Complex 3D Surfaces:** Create materials and techniques that allow for the fabrication of electronic components with consistent and predictable properties on intricate 3D surfaces.
(3) **Understanding Implant Structure Dynamics:** Investigate cell proliferation, degradation, and failure mechanisms within implant structures to gain a deeper understanding of their behavior and optimize their performance.
(4) **Harnessing Body Resources:** Explore the utilization of the body's resources, such as bio currents, motion, and temperature, as a continuous power supply for electronic components, reducing the need for external power sources or batteries.
(5) **Clinical Significance and Viability:** Evaluate the clinical significance, risks, and economic feasibility of developing more sophisticated structures for smart implants, taking into account factors like patient outcomes, safety considerations, and cost-effectiveness.

Addressing these challenges will pave the way for advancements in smart implant technology and contribute to improving patient outcomes in various biomedical applications.

## 7 Conclusions

Smart implants hold tremendous promise in enhancing medical care and patient outcomes. These cutting-edge devices provide real-time access to critical health information related to implant conditions and recovery progress. We offer a unified roadmap for smart implants to summarize all the challenges and issues. This roadmap will allow us to assess promising areas for the development of improved technologies for smart implants for their widespread use in clinical practice. Our analysis identifies four main directions for improving current smart implant technologies: material biocompatibility, wireless data transmission, long-term stable operation, and reliable power supply. To address material biocompatibility, research should focus on developing materials that promote integration and minimize adverse reactions. Reliable and efficient wireless communication protocols are crucial to ensure seamless and secure data transmission. The long-term stable operation can be enhanced by preventing the accumulation of biological contaminants on the sensitive implant's components by developing antibiofouling materials or coatings. The power supply can be improved through robust power management systems and optimized power consumption. Our comprehensive review of different implant types used for remote monitoring highlights these devices' opportunities and limitations.

With continued investment and progress in this field, we have high hopes for the widespread adoption of smart implants in the near future. They have the potential to revolutionize the way healthcare is delivered and received, bringing us closer to a future of personalized and effective medical treatment accessible to all individuals.

## Declaration of competing interest

The authors declare that they have no known competing financial interests or personal relationships that could have appeared to influence the work reported in this paper.

## Data Availability

This is a review article. Data used are cited in the text
